# The Influence of Social Support on Hematopoietic Stem Cell Transplantation Survival: A Systematic Review of Literature

**DOI:** 10.1371/journal.pone.0061586

**Published:** 2013-04-18

**Authors:** Sara Beattie, Sophie Lebel, Jason Tay

**Affiliations:** 1 School of Psychology, University of Ottawa, Ottawa, Ontario, Canada; 2 Ottawa Hospital Research Institute, Ottawa, Ontario, Canada; 3 Department of Medicine, University of Ottawa, Ottawa, Ontario, Canada; University of California, United States of America

## Abstract

**Background:**

Hematopoietic Stem cell Transplantation (HSCT) can negatively impact the psychosocial well-being of the patient. Social support is a complex term that has been variably used to encompass perceived and objective support, including caregiver presence. Social support has been associated with superior psychosocial outcomes; however the influence of social support on HSCT survival remains unclear. We sought to summarize the literature on the influence of social support on HSCT survival.

**Methods:**

Medline, EMBASE, Cochrane, CINAHL, and PsycINFO were searched using the following search categories/concepts: 1) HSCT, 2) Social support, 3) Caregiver, 4) Survival, and 5) Treatment outcomes.

**Results:**

We identified 6 relevant studies: 4 publications, 1 dissertation, and 1 abstract. Three studies were retrospective and 3, prospective. Sample size ranged between 92–272 with a mean/median patient age between 30–55 yrs. The duration of follow-up ranged between 13.3–48 months. Social support was measured inconsistently: 2 by retrospective investigator assessment, 2 as patients’ perceived support, 1 as caregiver presence, and 1 included caregiver presence and retrospective investigator assessment. The 4 published studies and 1 abstract demonstrate an association between better social support and survival. However, the unpublished dissertation, with the largest sample size found no association.

**Conclusions:**

There is a paucity of evidence examining social support with HSCT survival. Available studies are older, with the most recent publication in 2005. A heterogeneous group of HSCT patients were studied with variable follow-up times. Further, covariates were variably considered in HSCT survival analyses and we suggest that there may be publication bias, given the negative unpublished study with the largest sample size. Prospective studies using validated scales are necessary to better assess the influence of social support on HSCT mortality. Given the potential for improved HSCT survival with better social support, HSCT centres should routinely provide HSCT recipients and their caregivers with enhanced psychosocial services.

## Introduction

The outcomes of patients undergoing hematopoietic stem cell transplant (HSCT) are dependent on a multitude of variables such as transplant type, underlying disease and stability, as well as psychosocial factors [1]. Current literature suggests that the presence and quality of social support may provide meaningful benefits in quality of life [2] as well as in multiple psychological domains in the HSCT recipient [3], [4]. Specifically, higher levels of social support have been associated with improved physical and emotional well-being [5] and lower levels of distress and post-traumatic stress symptoms [6], [7]. Recently, extended family support and formal peer support has been associated with decreased treatment delays in a group of lymphoma patients that were considering undergoing HSCT [8]. On the other hand, low levels of social support have been associated with increased depressive symptoms [9] and post-traumatic stress symptoms [7]. Indeed, these psychological benefits often persist beyond the acute phases of HSCT and contribute to the overall quality of life for the HSCT recipient [10]. Social support is a variable that is often researched with psychosocial outcomes; however there have been few studies that examine the impact of social support on survival.

Social support is a complex term that has been variably measured, including self-report questionnaires of perceived and objective support, as well as network indicators such as presence of a spouse or a caregiver. Healthcare teams often rely on patients’ social support and particularly on family caregivers to provide informal care-giving, including medication administration, transportation to and from the hospital, and psychological support. This is particularly salient for HSCT programs that have shifted to performing outpatient HSCTs where the presence of a primary caregiver is required.

Despite increasing reliance on informal care-giving and recent data suggesting that the presence and quality of social support may improve HSCT survival, social support and/or presence of a caregiver are seldom assessed and take into account by the health care team. We therefore sought to summarize the literature on the influence of social support on HSCT survival and to identify any factor(s) that could contribute to the effect of social support on survival.

## Methods

We performed a search on the following databases using the OVID interface on 10 July 2012∶1) MEDLINE (1950 to July week 1, 2012), 2) EMBASE (1980 to 2012 week 29), 3) CINAHL (1981 to 10 July 2012), 4) PsycINFO (1806 to July week 4, 2012) and 5) All EBM reviews (1950 to the second quarter of 2012) using the search strategy detailed in Figure 1. Specifically, the following search categories/concepts were used: 1) Hematopoietic Stem Cell Transplantation, 2) Social Support, 3) Caregiver/Spouse, 4) Survival and 5) Treatment Outcomes including survival. Any potential “grey” literature was sought using Google Scholar. Further, local clinicians were approached to help identify any additional studies as well as a review of included article references.

A priori, our sought primary outcome was overall survival, while our secondary outcomes include transplant related mortality, acute and chronic graft versus host disease, and infections. These “hard” outcomes are commonly reported in HSCT literature [11]. Studies that met the following inclusion criteria were included in our review: 1. Patients >18 years undergoing hematopoietic stem cell transplantation, 2. Compared the presence of and/or quality of social support, and 3. Measured at least our primary study outcome of overall survival. Published studies, abstracts and dissertations were included. We excluded non-English studies, pediatric studies, and studies that did not report on at least a survival endpoint.

Two reviewers (SB and JT) independently applied the inclusion and exclusion criteria to the articles identified by the search strategy and extracted the data using a standardized data extraction form. The extraction included details on the publication source, demographics of the patients and clinical outcomes. Full text articles assessed for inclusion were discussed between the two reviewers. Discrepancies were adjudicated by a third party (SL). Finally, study quality was assessed using the Newcastle-Ottawa Scale (NOS) [12].

## Results

Our search systematic search strategy identified a total of 853 potential articles from five databases, with 6 potential articles identified from other sources. Two hundred and fifty duplicate articles were excluded and the remaining 609 were screened for relevance based on their titles and abstracts. Of these, 15 were deemed potentially eligible and retrieved for full review. After detailed review, 9 of these articles were excluded for the following reasons: Two articles were review articles, 2 articles did not specifically assess HSCT patients and the remaining 5 articles did not evaluate social support and/or caregiver presence as an a priori analytic plan. Subsequently, 6 articles met our inclusion criteria and were included in our systematic review (Figure S1). Further, we did not identify any articles that reviewed presence of and/or quality of social support with either transplant related mortality, acute and chronic graft versus host disease or infections. There were no discrepancies between the 2 reviewers with regard to studies chosen for inclusion. Given the heterogeneity of the included articles, a formal meta-analysis was not performed.

### Study Demographics

Our search identified 6 relevant studies (Table S1): 4 publications [13]–[16], 1 dissertation [17], and 1 abstract [18]. Five studies were conducted in USA [14]–[18] with one study from Germany [13]. All 6 studies were from single institutions at academic centres. Three studies were retrospective [14]–[16] and 3, prospective [13], [17], [18]. The study sample sizes ranged between 92–272, with either a mean or median patient age between 30–55 years. The duration of follow-up ranged between 13.3–48 months. One study assessed exclusively autologous HSCT patients [14] while 3 studies assessed exclusively allogeneic HSCT patients [14], [16], [18]. There were 2 studies that reviewed both allogeneic and autologous HSCT patients [15], [17].

### Study Measures of Social Support

Confidence in the results of identified studies is optimized by prospectively conducted studies using well validated scales, with excellent psychometric properties as well as assessments carried out by a dedicated trained investigative team. In our review, our identified studies assessed social support either by 1) validated scales [13], [17], 2) investigator rating [14]–[16], or 3) physical presence of a caregiver [16], [18].

Two studies evaluated social support by retrospective investigator assessment [14], [15]. Colon et al. performed a retrospective chart review, specifically looking for reports on patient’s perception of “extent of support received or anticipated from friends, spouse or significant other, and family” [14]. The investigators categorized the extent of perceived support subjectively as “high” or “low” where it appeared the authors predominantly considered emotional support as part of this rating. There was no description on the individuals who assigned the ratings or the consistency of the ratings. Similarly, Rodrigue et al. performed a retrospective review of their patient’s psychological record [15]. The investigators subsequently assigned ratings using a 5 point Likert-type scaled for the following constructs: 1) affective functioning, 2) compliance, and 3) social support stability, which they combined into a single composite measure of “psychological variables”. Importantly, the investigators applied a detailed guide sheet and coding instructions, to ensure consistency of rating. Indeed, 2 trained “blinded” coders assessed inter-rater reliability and suggest that that their reliability was over 76%. Finally, the authors suggest that the constructs within their composite scale significant correlated with validated measures of SF-36 [19] and TSFQ [20].

Two studies evaluated patients’ perceived social support prospectively [13], [17]. Frick et al. utilized the Illness Specific Scales of Social Support (ISSS) that was designed for patients receiving a HSCT [13]. They specifically used 2 subscales: 1) perceived positive social support and 2) problematic support, which they state showed “satisfactory reliability and construct validity”, but did not provide further details. In contrast, Artherholt et al. prospectively assessed perceived social support using the Social Support Inventory subscale: Satisfaction with Social Support received [17]. This validated 7 point Likert-like scale (Cronbach α between 0.78–0.93) assesses both the extent of sought and received support, together with satisfaction with received support from 3 types of supporters: 1) family or friend, 2) care provider and 3) bone marrow donor. A composite score is subsequently derived from the 3 elements.

The study by Foster el al. evaluated both caregiver presence and social support [16]. Specifically, the investigators performed a retrospective chart review to ascertain the presence or absence of a lay-partnered caregiver. They defined a lay-partnered caregiver as “one person being present during the patient’s hospital stay seven or more hours a day, five or more days a week, beginning of the day of admission”. They also assessed and assigned the adequacy and efficacy of social support by an investigator created 4 point Likert-like scale. There was no clear documentation on the person(s) performing the ratings or the reliability of the assigned ratings. Similarly, the abstract by McLellan et al. prospectively evaluated the presence or absence of a lay partnered of caregiver [18]. Ascertainment of the caregiver was not described in the abstract.

### Study Outcomes-Mortality

The 4 published studies [13]–[16] and 1 abstract [18] demonstrate a statistical association between better social support and survival (Table S2). However, the unpublished dissertation [17], with the largest sample size found no statistical association. The covariates considered by the 6 identified studies varied between studies when assessing the association between social support and survival. Formal power calculations were not provided by the identified studies. Further, none of the identified studies reported on other morbidity outcomes such as graft-versus host disease, infectious complications or length of hospital stay. Consequently, a formal meta-analysis was not performed given the heterogeneity of the data.

Colon et al. suggests “high”, as compared with “low”, social support was associated with improved overall survival at 24 months (55% and 20% respectively; p = 0.02) [14]. Further, the authors considered the following covariates in their analyses: 1) age, 2) gender, 3) marital status, 4) employment status, 5) education, 6) psychiatric history, 7) first degree relative psychiatric history, 8) psychiatric history at pre-transplant evaluation, 9) prominent symptoms at pre-transplant evaluation: depressed, anxious, 10) subtype of acute leukemia, 11) illness stage and 12) year of HSCT. Nonetheless, social support remained independent and statistically associated with survival.

With a median follow-up of 20.3 months, Rodrigue et al. determined that social support stability was independently and significantly associated with survival (p<0.05) where they considered social support together with the following covariates: 1) disease risk status, 2) quality of graft match and 3) affective functioning within a logistic regression model. Odds ratios were not provided for the potential influence of social support [15].

Frick et al. performed a multivariate survival analysis, with the following covariates: 1) positive social support, 2) problematic social support, 3) received psychotherapy, 4) Karnofsky performance status, 5) interferon use, 6) depression, 7) age, 8) marital status and 9) diagnosis [13]. They demonstrated that problematic social support was independently associated with survival (RR 3.649; 95%CI: 1.282–10.382).

Similarly, Foster el al. performed a multivariate stepwise survival analysis [16]. They considered the following as potential risk factors for survival: 1) gender, 2) race, 3) marital status, 4) religion, 5) living arrangements, 6) patient’s social supports, 7) spirituality as support, 8) patient’s coping, 9) family’s coping, 10) history of smoking/substance abuse or psychiatric diagnosis, 11) presence of advanced directive, 12) presence of lay partnered caregiver, 13) disease diagnosis, 14) disease status, 15) type of HSCT donor and 16) presence of graft versus host disease. The presence of a lay-partnered caregiver was associated with survival (RR 3.40; 95%CI: 2.14–5.38).

In their abstract, McLellan et al. state that survival rates were significantly associated with a consistent lay partner duration of >3 hours (p = 0.004) and frequency of partner visits >75% of inpatient days (p = 0.004) [18]. Further, the presence of a lay partnered caregiver was associated with a 42% survival as compared to 26% in those without caregivers.

Artherholt et al. state that there is no statistical association between social support and HSCT mortality but a specific p value or relative risk was not provided [17]. In their analyses, they performed a Cox proportional hazard model to assess mortality and potential predictors of mortality. They included the following covariates: 1) age, 2) gender, 3) type of HSCT, 4) previous radiation or chemotherapy, 5) physical complications pre-transplant as measured by the Sickness Impact Profile Physical Subscale score, 6) disease risk category and 7) presence and extent of graft versus host disease.

### Quality of Studies

All included studies scored well using the Newcastle-Ottawa Quality Assessment Scale for Cohort Studies (Table S3) [13]–[17]. Only 1 study, the abstract, did not receive the maximum allowable score for comparability [18].

## Discussion

Our review suggests that there is a paucity of evidence examining social support with HSCT survival. Available studies are older, with the most recent publication in 2005. Social support was inconsistently defined and measured among a heterogeneous group of HSCT patients with variable follow-up times. Further, covariates such as disease status prior to transplant known to influence HSCT survival were variably considered in analyses. Importantly, the concepts and/or elements that aimed to encompass social support differed between the identified studies. Although, the majority of the identified studies suggest a significant statistical association between social support and overall survival, the largest unpublished study did not demonstrate this association.

The underlying variables that explain the potential positive influence of social support on HSCT survival need to be identified. Potential components of social support may include actual as well as perceived 1) instrumental support, and 2) emotional support. Instrumental support such as enhanced medication compliance and adherence, timely and frequent access to healthcare has been considered as the reason for social support's effect. It could be argued that instrumental support is paramount within a program that focuses on outpatient based HSCT, however, evidence supporting its effect is varied [21], [22]. For instance, in patients receiving HSCT Rodrigue et al. determined that social support, but not patient compliance determined superior overall survival [15]. Consequently, the authors postulate that the importance and influence of social support may be determined by the stability of social network as opposed to only the presence of social support enhancing patient compliance. This would suggest that positive influence of social support on survival is attributed to more than instrumental support. Thus, emotional support, such as social attachment, feeling able to discuss challenging decisions or fears with another person(s) may also be a relevant component of social support [23]. Indeed, Colon et al. demonstrate that “high” as compared to “low” perceived social support was associated with improved survival at 24 months (55% and 20% respectively; p = 0.021) [14]. Subsequently, the authors hypothesize that social support may serve as a buffer leading to better coping, improved mood and consequently better health outcomes. Additionally, the construct of social support may incorporate elements of “finding meaning” and this concept has been associated with improve immune function leading to positive clinical outcomes [24].

Previous research in other oncology populations has demonstrated variable evidence for both instrumental and emotional support. Lutgendorf et al. found that amongst women with ovarian cancer, those with higher social attachment had a survival advantage whereas patient survival was not associated with level of instrumental support [21]. On the other hand a study with acute myeloid leukemia patients found that although higher instrumental and higher emotional social support were univariate predictors of lower mortality, when they were considered simultaneously, only instrumental social support was associated with mortality [22]. Indeed, there have been suggestions that emotional support has been correlated with superior immunity, cardiovascular and endocrine function ultimately leading to improved mortality outcomes [21]–[23], [25]. Taken together, the relative importance of instrumental and emotional support remains unclear not only in HSCT, but in general oncology. Perhaps both aspects of social support are necessary in order to maximize patient outcomes or that instrumental support is of utmost importance early in the HSCT while emotional support plays an increasing role later in the HSCT trajectory.

Although most of the current research has investigated whether the presence of social support has either a negligible or positive effect on HSCT mortality, the quality of social support may be the important factor that impacts HSCT mortality. This concept was investigated by only one study to date: Frick et al [13]. Indeed, they state that social support is a “double-edged sword which can also be maladaptive and have a negative effect…” In contrast to the other studies in this review, the authors demonstrate that positive social support does not impact HSCT survival. However, the presence of problematic social support, as measured by the Illness Specific Scales of Social Support was associated with inferior survival [13]. This suggests that not all forms of social support are beneficial and in fact having a support system that includes critical, invalidating, and pessimistic others may in fact negatively influence HSCT survival. The results of this study further support the notion that the potential benefit of social support may be more attributable to emotional support rather than instrumental support.

A partnered caregiver could be considered the optimal “intervention” that embraces both emotional and instrumental support. To date, only one study by Foster et al. distinguished partnered lay caregivers from the support of multiple family members - “general” social support [16]. Specifically, they emphasize that “general” social support lacks “the interpersonal resonance and the interactive empathy characteristic of partnered relationships.” Not surprisingly, the presence of a partnered caregiver correlates with better social support. However, the authors were able to demonstrate that an absence of a partnered lay caregiver was independently associated with inferior overall survival (HR 3.06 95%CI 1.92–4.88) while social support was not. Interestingly, this risk of inferior overall survival associated with absent partnered lay caregiver is even higher than traditional variables such as related or unrelated donor HSCT (HR 2.39) as well as disease status-active/remission (HR 1.85). In their abstract, McLellan et al. subsequently confirms these findings in their prospective cohort of patients where the 4 year survival of allogeneic HSCT recipients with partnered caregivers compared to those without were 42% and 26% respectively (p = 0.004) [18].

There are limitations to our review that deserves attention. Firstly, there appears to be a potential for publication bias within our review, given the negative unpublished study with the largest sample size. Indeed, there may be other clinical studies that were not identified by our review leading to a “file drawer” effect. However, our rigorous search strategy across multiple databases aimed to limit this possibility. Second, the heterogeneity of the study design, assessments and analyses as previously discussed precludes any formal meta-analysis. Specifically, a clear definition of social support in the identified studies was seldom provided and its measurement varied and not necessarily standardized. Nonetheless, our review enabled us to identify and summarize the most current available evidence on social supports influence on mortality post-HSCT.

We believe that the influence of social support on survival is an increasingly important but neglected area of investigation in HSCT research. Two studies suggest that the presence of a consistent caregiver in patients undergoing allogeneic HSCT improves survival outcomes [16], [18]. Also, the quality of social support has been associated with superior survival outcomes [13]; however the relative effect of caregiver presence to other forms of social support on survival remains unclear. These intriguing findings need to be confirmed by larger and more rigorous studies before firm conclusions can be made. We suggest that future research in this area should provide a clear definition of social support and its measurement using standardized and validated scales [26], [27]. If these findings are indeed confirmed, further understanding the influence of both actual and perceived social support as well as the components associated with its influence will help optimize HSCT recipients care. This includes better understanding the elements(s) that constitute social support, for example, if the presence of a primary caregiver is what adds a survival benefit or it is a patients’ overall sense of global social support.

Caregivers of HSCT patients are known to experience significant psychosocial sequelae during the transplant trajectory, including elevated emotional distress, burden and fears about the future [28]. Given the potential for presence of a partnered caregiver to positively influence HSCT recipient’s psychosocial outcomes and perhaps survival, it is imperative that psychological supports be available for the caregiver. Providing caregivers and loved ones with psycho-education on the care-giving process as well as psychosocial support will enhance the psychological health of both patients and caregivers. Beyond psychological health, the improved quality of social support may potentially improve the survival rates of HSCT patients. In the absence or lack of social support, the extended health care team should consider providing the more vulnerable HSCT recipient with enhanced psychosocial services.

## Supporting Information

Figure S1 **Flow diagram summarizing the identification process of relevant clinical trials.**
(DOCX)

Table S1 **MEDLINE Search Strategy.**
(DOCX)

Table S2 **Summary of Identified Studies.** *Dissertation; +Abstract; Auto: Autologous; Allo: Allogeneic; SS: Social Support; CG: Caregiver; NR: not reported; NA: not applicable
(DOCX)

Table S3 **Newcastle-Ottawa Quality Assessment Scale for Cohort Studies** [**12**] **.** Note: A study can be awarded a maximum of one star for each numbered item within the Selection and Outcome categories. A maximum of two stars can be given for Comparability
(DOCX)
