# Correction: The Influence of Social Support on Hematopoietic Stem Cell Transplantation Survival: A Systematic Review of Literature

**DOI:** 10.1371/annotation/9e6ee407-7782-4cf7-8e82-9f38c1d6739e

**Published:** 2013-05-07

**Authors:** Sara Beattie, Sophie Lebel, Jason Tay

The figures and tables in the Supporting Information section should have been included in the main text of the article. They were instead included as Supporting Information files, and thus not present in the PDF. Figures and tables can be viewed and downloaded from the Supporting Information section. In the first paragraph of the Methods section, the authors' reference to Figure 1 should be to Figure S1.

The PRISMA Checklist for Systematic Reviews/Meta-analyses was omitted. The PRISMA Checklist can be viewed in two parts here:

Part 1: 

**Figure:**
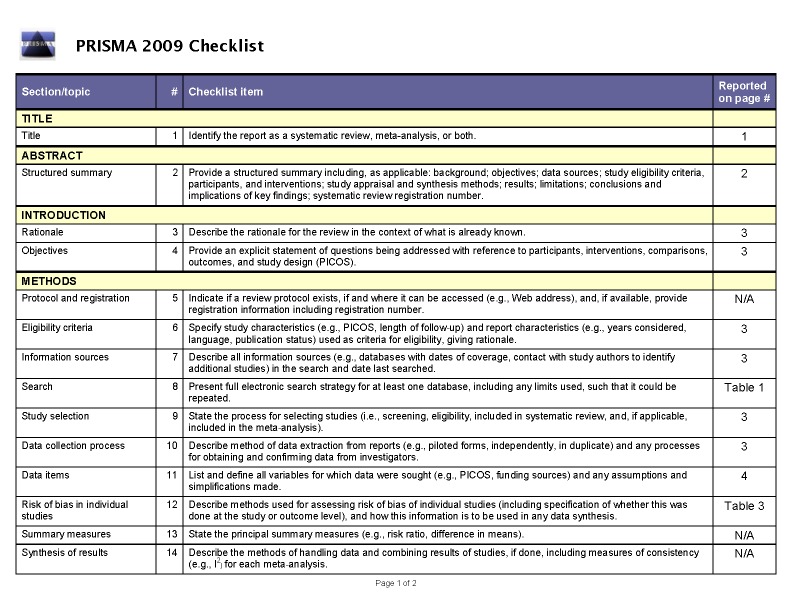


[^]

Part 2: 

**Figure:**
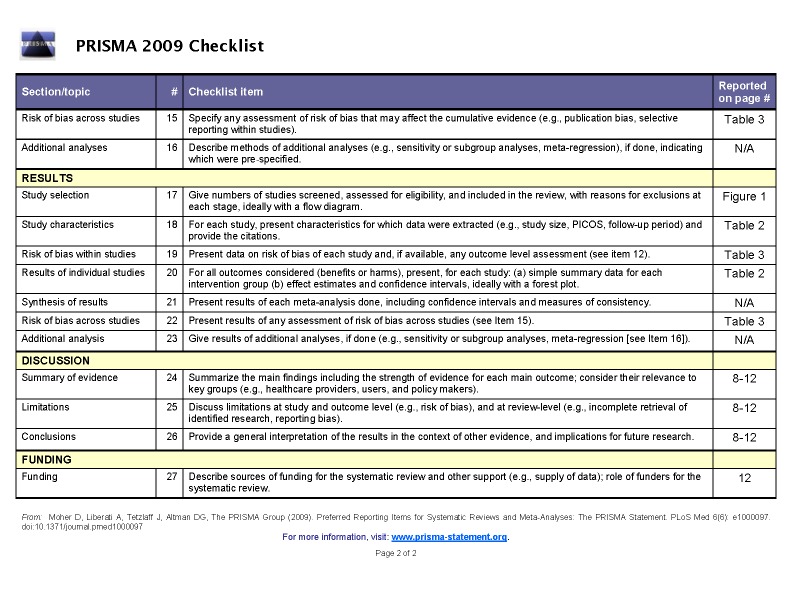


[^] 

