# Supplementary material for: The Influence of Social Support on Hematopoietic Stem Cell Transplantation Survival: A Systematic Review of Literature
Source: PLoS One. 2013 Apr 18;8(4):e61586. doi: 10.1371/journal.pone.0061586 (PMC3630123; doi:10.1371/journal.pone.0061586)
Supplement: Figure S1 — Flow diagram summarizing the identification process of relevant clinical trials. (DOCX) [file pone.0061586.s001.docx]

Figure S1.  Flow diagram summarizing the identification process of relevant clinical trials
Full-text articles excluded (n = 9):		2 review articles		5 articles did not primarily evaluate social support and/or caregiver presence		2 articles did not assess HSCT patientsFull-text articles excluded (n = 9):		2 review articles		5 articles did not primarily evaluate social support and/or caregiver presence		2 articles did not assess HSCT patientsAdditional records identified through other sources
(n = 6)Additional records identified through other sources
(n = 6)Studies included in quantitative synthesis (meta-analysis)
(n = 0)Studies included in quantitative synthesis (meta-analysis)
(n = 0)Studies included in qualitative synthesis
(n = 6)Studies included in qualitative synthesis
(n = 6)Records excluded
(n = 594)Records excluded
(n = 594)Full-text articles assessed for eligibility
(n = 15)Full-text articles assessed for eligibility
(n = 15)Records screened
(n = 609)Records screened
(n = 609)Records after duplicates removed
(n =609)Records after duplicates removed
(n =609)Records identified through database searching:		Medline 	(n = 136)		EMBASE 	(n = 299)		Cochrane 	(n = 43)		PsyINFO 	(n = 145)		CINAHL 	(n = 230)Records identified through database searching:		Medline 	(n = 136)		EMBASE 	(n = 299)		Cochrane 	(n = 43)		PsyINFO 	(n = 145)		CINAHL 	(n = 230)


		
