# Supplementary material for: The Influence of Social Support on Hematopoietic Stem Cell Transplantation Survival: A Systematic Review of Literature
Source: PLoS One. 2013 Apr 18;8(4):e61586. doi: 10.1371/journal.pone.0061586 (PMC3630123; doi:10.1371/journal.pone.0061586)
Supplement: Table S1 — MEDLINE Search Strategy. (DOCX) [file pone.0061586.s002.docx]

**Table S1: MEDLINE Search Strategy**

| 1. Hematopoietic Stem Cell Transplantation/ |
| --- |
| 1. h?ematopoietic stem cell transplant$.tw. |
| 1. (hsct or h?ematopoietic sct).tw. |
| 1. stem cell transplant$.tw. |
| 1. Peripheral Blood Stem Cell Transplantation/ or pbsct.tw |
| 1. (peripheral blood cell transplant$ or peripheral blood stem cell transplant$ or peripheral stem cell transplant$).tw. |
| 1. Bone Marrow Transplantation/ or (bone marrow transplant$ or bmt).tw. |
| 1. blood transplant$.tw. |
| 1. ((autologous or allogeneic or allogenic) adj2 (transplant$ or graft$)).tw. |
| 1. or/1-9 |
| 1. social support/ |
| 1. ((social or partner or spousal or family or peer or emotional or instrumental) adj support).tw. |
| 1. (social adj (contact or relationships or isolation or networks or connections)).tw. |
| 1. Caregivers/ or caregiver$.tw. |
| 1. Spouses/ or spouse$.tw. |
| 1. or/11-15 |
| 1. Survival/ or Survival Rate/ or Survival Analysis/ |
| 1. survival.tw. |
| 1. Treatment Outcome/ |
| 1. outcome$.tw. |
| 1. "Quality of Life"/ or "quality of life".tw. |
| 1. or/17-20 |
| 1. 10 and 16 and 22 |
