# Supplementary material for: The Influence of Social Support on Hematopoietic Stem Cell Transplantation Survival: A Systematic Review of Literature
Source: PLoS One. 2013 Apr 18;8(4):e61586. doi: 10.1371/journal.pone.0061586 (PMC3630123; doi:10.1371/journal.pone.0061586)
Supplement: Table S2 — Summary of Identified Studies. *Dissertation; +Abstract; Auto: Autologous; Allo: Allogeneic; SS: Social Support; CG: Caregiver; NR: not reported; NA: not applicable (DOCX) [file pone.0061586.s003.docx]

**Table S2: Summary of Identified Studies**

| **Authors** | **Type of**  **Transplant** | **Follow-up Duration** | **Participants**  **(n)** | **Gender** | **Age**  **(years)** | **Social support/**  **Caregivers** | **Comparisons/**  **Social Support (SS) Scales used** | **Unadjusted Survival** | **Positive Study** | **Adjusted Survival** | **Authors' comments and/or proposed Hypothesis of Effect** |
| --- | --- | --- | --- | --- | --- | --- | --- | --- | --- | --- | --- |
| Artherholt 2007* | 83% Allo  17% Auto | 400  days | 272 | 56% Males | Mean 36 | NA | SS Inventory subscale (satisfaction with SS received) | NR | no | yes | 1. The medical factors such as disease status "overwhelmed" any potential predictive potential of SS. 2. Superior SS may be associated with concept of "Finding meaning" in their diagnosis, leading to improved HSCT outcomes. 3. Positive meaning finding has been associated with a better immune function. |
| Colon  1991 | Allo | 24  months | 100 | 65% Males | Mean 30 | High SS: 29;  Low SS 71 | Investigator rating of SS based on spouse/family | 55% vs. 20%  (at 24 months) | yes | yes | 1. SS serves as a buffer leading to better coping, improved mood and greater attention to required SS. This leads to better health outcomes. |
| Frick  2005 | Auto | NR | 99 | 57% Males | Median 55 | Problematic SS vs. No problematic SS | Illness specific Scales  of SS | 78% vs. 40%  (at 47 months) | yes | yes | 1. Positive SS does not impact HSCT survival, but rather the presence of problematic (negative) SS was associated with inferior survival. |
| Foster  2004 | Allo | 48  months | 131  (CG: 72;  No CG:59) | 60% Males | NR | 61% spouses;  31% parents;  9% others | Absence/presence  of caregiver and Investigator rating of SS | 54% vs.15% | yes | yes | 1. Presence of a lay partnered caregiver was independently correlated with superior survival. 2. The "elements" of a caregiver remains elusive, and given the absence of understanding it would impossible to replicate its effect in practice |
| McLellan  2011^+^ | Allo | 48  months | 164  (CG: 88;  No CG: 76) | NR | Median 44 | 60% spouses;  30% parents;  10% others; | Absence/presence  of caregiver | 42% vs. 26% | yes | NR | 1. Not discussed |
| Rodrigue  1999 | 54% Auto  46% Allo | 20.3 months | 92 | 53% Males | Mean 36.3 | NA | Investigator rated of SS using a Likert scale | NR | yes | yes | 1. SS, but not patient compliance determined superior overall survival. 2. SS may be determined by the stability of social network as opposed to only instrumental support. |

**Legend for Table S2: Summary of Identified Studies**

*Dissertation;

+Abstract;

Auto: Autologous;

Allo: Allogeneic;

SS: Social Support;

CG: Caregiver;

NR: not reported;

NA: not applicable
