# Supplementary material for: The Influence of Social Support on Hematopoietic Stem Cell Transplantation Survival: A Systematic Review of Literature
Source: PLoS One. 2013 Apr 18;8(4):e61586. doi: 10.1371/journal.pone.0061586 (PMC3630123; doi:10.1371/journal.pone.0061586)
Supplement: Table S3 — Newcastle-Ottawa Quality Assessment Scale for Cohort Studies [12] . Note: A study can be awarded a maximum of one star for each numbered item within the Selection and Outcome categories. A maximum of two stars can be given for Comparability (DOCX) [file pone.0061586.s004.docx]

**Table S3: Newcastle-Ottawa Quality Assessment Scale for Cohort Studies [12]**

| **AUTHORS** | **SELECTION** | | | | ***COMPARABILITY*** | ***OUTCOME*** | | |
| --- | --- | --- | --- | --- | --- | --- | --- | --- |
|  | *Representativeness of the Exposed Cohort* | *Selection of the Non-Exposed Cohort* | *Ascertainment of Exposure* | *Demonstration That Outcome of Interest Was Not Present at Start of Study* | *Comparability of Cohorts on the Basis of the Design or Analysis* | *Assessment of Outcome* | *Was Follow-Up Long Enough for Outcomes to Occur* | *Adequacy of Follow Up of Cohorts* |
| **Artherholt**  **2007*** | 🟑 | 🟑 | 🟑 | 🟑 | 🟑🟑 | 🟑 | 🟑 | 🟑 |
| **Colon**  **1991** | 🟑 | 🟑 | 🟑 | 🟑 | 🟑🟑 | 🟑 | 🟑 | 🟑 |
| **Frick**  **2005** | 🟑 | 🟑 | 🟑 | 🟑 | 🟑🟑 | 🟑 | 🟑 | 🟑 |
| **Foster**  **2004** | 🟑 | 🟑 | 🟑 | 🟑 | 🟑🟑 | 🟑 | 🟑 | 🟑 |
| **McLellan**  **2011^+^** | 🟑 | 🟑 | 🟑 | 🟑 |  | 🟑 | 🟑 | 🟑 |
| **Rodrigue**  **1999** | 🟑 | 🟑 | 🟑 | 🟑 | 🟑🟑 | 🟑 | 🟑 | 🟑 |

**Legend for Table S3: Newcastle-Ottawa Quality Assessment Scale for Cohort Studies [12]**

Note: A study can be awarded a maximum of one star for each numbered item within the Selection and Outcome categories. A maximum of two stars can be given for Comparability
